# GABAA agonist muscimol ameliorates learning/memory deficits in streptozocin-induced Alzheimer’s disease non-transgenic rat model

**DOI:** 10.1186/2193-1801-4-S1-P36

**Published:** 2015-06-12

**Authors:** Vladimirs Pilipenko, Jolanta Pupure, Juris Rumaks, Ulrika Beitnere, Zane Dzirkale, Raimonds Skumbins, Vija Klusa

**Affiliations:** Faculty of Medicine, Department of Pharmacology, University of Latvia, Latvia

**Keywords:** memory, muscimol, streptozocin

**Background:** GABAergic inhibitory action regulates learning/memory processes and contributes to neurotransmission (Gong et al., 2009). Existing evidence suggests GABAergic system is involved in pathophysiology of Alzheimer’s disease (AD) via inhibitory interneuron deficits (Verret et al., 2012) and decrease in functional GABAA receptors (Limon et al., 2012). In vitro, GABA and muscimol (GABAA receptor agonist) blocked neuronal death induced by Aβ in rat hippocampal and cortical neurons (Paula-Lima et al., 2005). Our concept: low doses of muscimol may prevent learning/memory deficits in intracerebroventricular (icv) streptozocin (STZ)-induced AD non-transgenic rat model.

**Methods.** Wistar male rats (280±20 g) were pre-treated with saline (control) or muscimol (0.01 and 0.05 mg/kg) for 3 days. On day 4, rats received icv STZ (100 μg/ml) or aCSF. From day 18, rats received muscimol or saline for 4 days; rat spatial learning and memory were assessed in water maze test (4 trials/day) by recording the time to reach the hidden platform (escape latency). A probe trial without platform was carried out 24 h after the training trials, and the number of platform zone crossings has been recorded.

**Results.** STZ statistically increased the escape latency vs. control group (p<0.0001). Muscimol at both doses significantly decreased the escape latency in STZ rats vs. STZ, reversing STZ effect by about 90% on days 3 and 4 (p<0.0001). In probe trial, the number of platform crossings in muscimol+STZ rats’ was significantly increased vs. STZ rats. Muscimol at both doses per se showed values comparable to control. Conclusions. Obtained data suggest that icv STZ significantly decreased rat spatial learning and memory and learning ability. Muscimol at both low doses significantly improved rats’ learning and memory abilities in both normal and AD-type rats. One may suggest that intensification of GABAergic processes may be a useful pharmacotherapeutic strategy to halt early memory decline in AD.

